# Influence of watershed characteristics on streambed hydraulic conductivity across multiple stream orders

**DOI:** 10.1038/s41598-020-60658-3

**Published:** 2020-02-28

**Authors:** Olufemi P. Abimbola, Aaron R. Mittelstet, Troy E. Gilmore, Jesse T. Korus

**Affiliations:** 1 Department of Biological Systems Engineering, University of Nebraska-Lincoln, 223 L. W. Chase Hall, Lincoln, NE 68583-0726 United States; 2 Conservation and Survey Division, School of Natural Resources, University of Nebraska-Lincoln, 101 Hardin Hall, 3310 Holdrege Street, Lincoln, NE 68583-0996 United States

**Keywords:** Environmental sciences, Hydrology

## Abstract

Streambeds are critical hydrological interfaces: their physical properties regulate the rate, timing, and location of fluxes between aquifers and streams. Streambed vertical hydraulic conductivity (*K*_*v*_) is a key parameter in watershed models, so understanding its spatial variability and uncertainty is essential to accurately predicting how stresses and environmental signals propagate through the hydrologic system. Most distributed modeling studies use generalized *K*_*v*_ estimates from column experiments or grain-size distribution, but *K*_*v*_ may include a wide range of orders of magnitude for a given particle size group. Thus, precisely predicting *K*_*v*_ spatially has remained conceptual, experimental, and/or poorly constrained. This usually leads to increased uncertainty in modeling results. There is a need to shift focus from scaling up pore-scale column experiments to watershed dimensions by proposing a new kind of approach that can apply to a whole watershed while incorporating spatial variability of complex hydrological processes. Here we present a new approach, Multi-Stemmed Nested Funnel (MSNF), to develop pedo-transfer functions (PTFs) capable of simulating the effects of complex sediment routing on *K*_*v*_ variability across multiple stream orders in Frenchman Creek watershed, USA. We find that using the product of *K*_*v*_ and drainage area as a response variable reduces the fuzziness in selecting the “best” PTF. We propose that the PTF can be used in predicting the ranges of *K*_*v*_ values across multiple stream orders.

## Introduction

Water scarcity is among the most pressing issues to humanity. Intensive water consumption, driven by a growing population and changing climate, places the world’s limited water supplies under increasing pressure. These stresses often propagate throughout a hydrologic system, because streams, rivers, and lakes are connected to underlying aquifers. For these reasons, the interaction between groundwater and surface water is of much interest to water managers. The water exchange or interaction pattern depends on substrate permeability^1–4^. *K*_*v*_ is one of the major parameters controlling stream-aquifer interactions. There are several reach-scale and watershed-scale variables which influence the spatial variation and distribution of *K*_*v*_ along and across stream reaches. These factors can be geological, hydrological, anthropogenic, or biological^5–11^. Some geologic factors that mostly influence streambed *K*_*v*_ are sediment particle size, underlying geology, heterogeneity of the substratum, thickness of bed material, channel geometry, hydraulic radius variations, and roughness due to natural and anthropogenic alterations^8^.

In hydrological modeling studies, homogeneity of *K*_*v*_ is usually assumed for practical reasons even though it may lead to more uncertainty in streamflow modeling^12,13^. Since it is not practical to measure *K*_*v*_ at every location along a stream course, modelers often rely on literature values or few measurements, and assume *K*_*v*_ does not vary across the watershed. Owing to lack of detailed information about the order of magnitude of its variation and the uncertainties in characterizing variability of *K*_*v*_, satisfactory results can be achieved, in many cases, by simply assuming that the streambed is homogeneous. However, assuming homogeneity across a watershed often leads to the under- or over-prediction of streambed leakage and baseflow^13–16^. It is imperative to reliably estimate the spatial distribution of *K*_*v*_ to improve hydrological models and better understand the connectivity between surface water and groundwater^17–20^.

There are many laboratory and *in-situ* tests to determine streambed *K*_*v*_^18,21–23^. While some studies focused on advantages and limitations of different measurement techniques^24,25^, others have focused on only the spatial variability of streambed *K*_*v*_ along transects across a channel^26^, both the spatial and temporal variability^18^, statistical description (means, ranges, variances) for hydraulic conductivity data^22,27^, or spatial interpolation of streambed *K*_*v*_^22,28^. These studies have all grappled with challenges of estimating *K*_*v*_ because of the difficulty of determining representative samples and comparing results, considering the heterogeneity and anisotropy of streambed materials and geological conditions^5^.

Similar to the issue of spatial variability is the effect of scaling. Based on some unsolved problems in hydrology that were recently published^29^, one of the unanswered questions posed was, “What are the hydrologic laws at the watershed scale and how do they change with scale?” They also questioned why dominant hydrological processes emerge and disappear across scales, and why hydrology seems to be simple at the watershed scale despite being complex at smaller scales^29^. Since the pore-scale approach to flow in porous media may be inherently inadequate at the watershed scale, a more fruitful path forward is to consider a watershed as a single ecosystem, and to build a new kind of theory or propose a new kind of approach that can apply to the whole watershed.

Saturated hydraulic conductivity (*K*_*sat*_) is a quantitative measure of the ability of a saturated soil to transmit water when subjected to a hydraulic gradient^30^. Although *K*_*v*_ and *K*_*sat*_ are similar in definition, the processes that govern their distribution are different. While *K*_*sat*_ is essential in modeling surface and subsurface flow as well as solute transport in soils and sediments^31^, *K*_*v*_ is an important variable controlling water and solute exchange between streams and surrounding groundwater systems^17–20^. Many pedo-transfer functions (PTFs) have been developed in the past five decades to estimate *K*_*sat*_ from easily measureable parameters, such as textural properties, bulk density, and sample dimensions^31–37^. In comparison to *K*_*sat*_, PTFs have not been as widely applied to streambed *K*_*v*_ estimation from other soil properties^38,39^. While most of the *K*_*v*_ studies have focused on analyzing the spatial and temporal variations of point measurements, very few empirical studies have focused on predicting *K*_*v*_ using only reach-scale attributes^8^. There is still a knowledge gap in the spatial prediction of streambed *K*_*v*_ using PTFs based on soil textural distribution and watershed characteristics. Although PTFs may not be applicable beyond the regions for which they were developed^39^, we attempted to develop PTFs for estimating *K*_*v*_ within a few orders of magnitude of the measurements using a new approach which hinges on the premise that the sediment composing the streambed is originated from the eroded rocks and sediment within its enclosing drainage area.

## Results and Discussion

The geometric mean *K*_*v*_ value varies between 7.57 × 10^−3^ and 1.81 m/day, about four orders of magnitude variation, which indicates different types of soils with various structures across different stream orders. The summary statistics of streambed *K*_*v*_ values at each of the ten test sites (stream channels) are shown in Table 1.

**Table 1:** Summary statistics of *K*_*v*_ (m/day) values at ten sites.

Site	Sample size, n	Geometric mean	Minimum	Maximum	Coefficient of variation	Skewness	Kurtosis
1	9	7.57 × 10^−3^	3.88 × 10^−3^	1.64 × 10^−2^	0.49	1.02	0.51
2	9	1.25 × 10^−2^	6.11 × 10^−3^	4.81 × 10^−2^	0.91	1.59	1.31
3	9	4.04 × 10^−2^	1.01 × 10^−2^	0.11	0.58	1.07	1.79
4	9	1.42	1.54 × 10^−2^	51.75	2.06	2.91	8.60
5	9	0.62	3.28 × 10^−3^	26.84	1.32	0.86	−1.36
6	9	8.66 × 10^−2^	5.65 × 10^−3^	0.45	0.96	1.66	2.84
7	12	0.16	3.03 × 10^−2^	1.13	1.12	2.27	6.03
8	9	2.23 × 10^−2^	4.87 × 10^−3^	0.19	1.36	2.49	6.71
9	9	0.10	4.48 × 10^−3^	0.78	1.11	0.91	−0.54
10	9	1.81	5.55 × 10^−2^	9.04	0.96	1.02	−0.39

**Figure 1:**
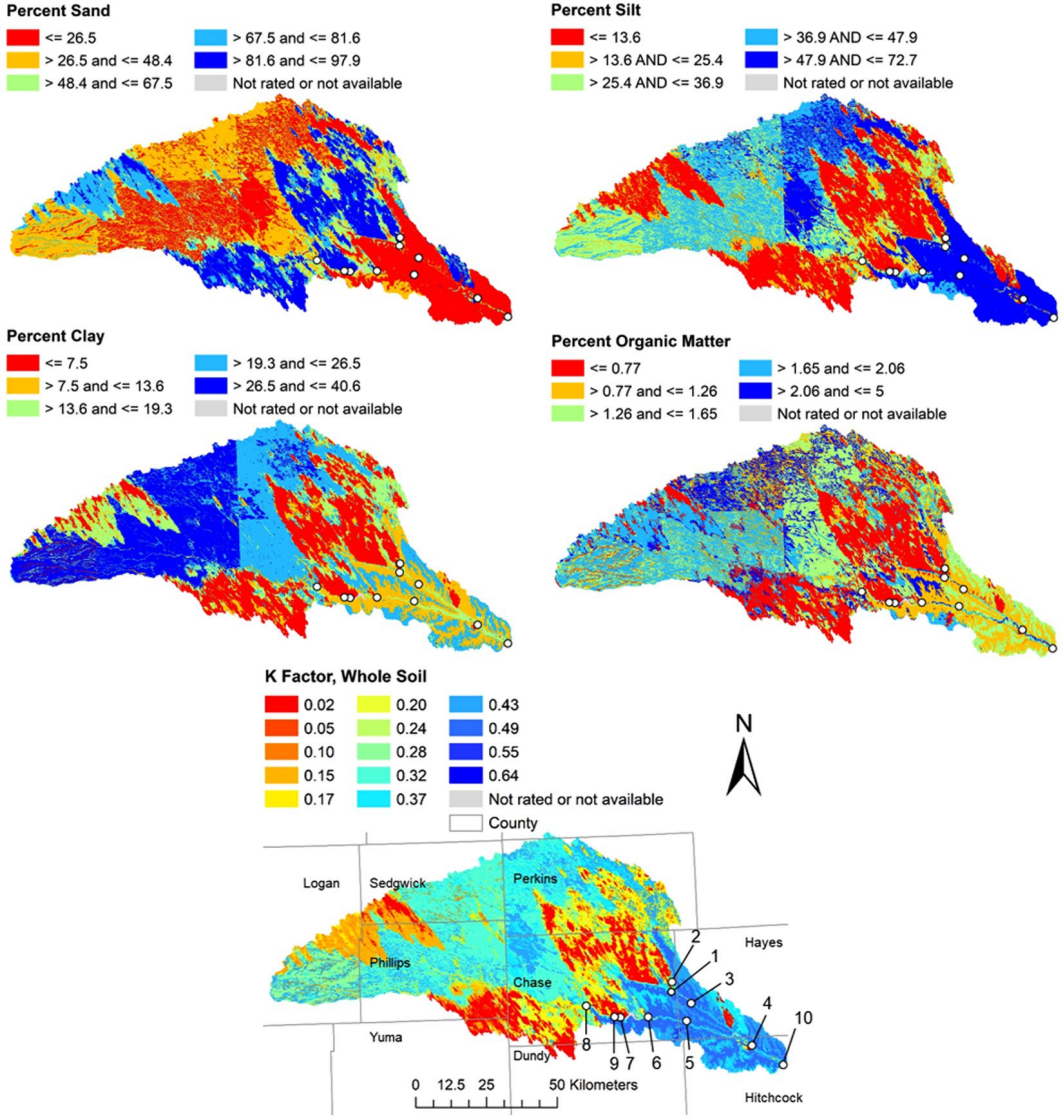
Spatial distributions of the soil properties for 0–50 cm depth. The sharp vertical and horizontal boundaries between classes in some maps are county boundaries which are effects of differences in how county soil surveys were conducted.

**Figure 2:**
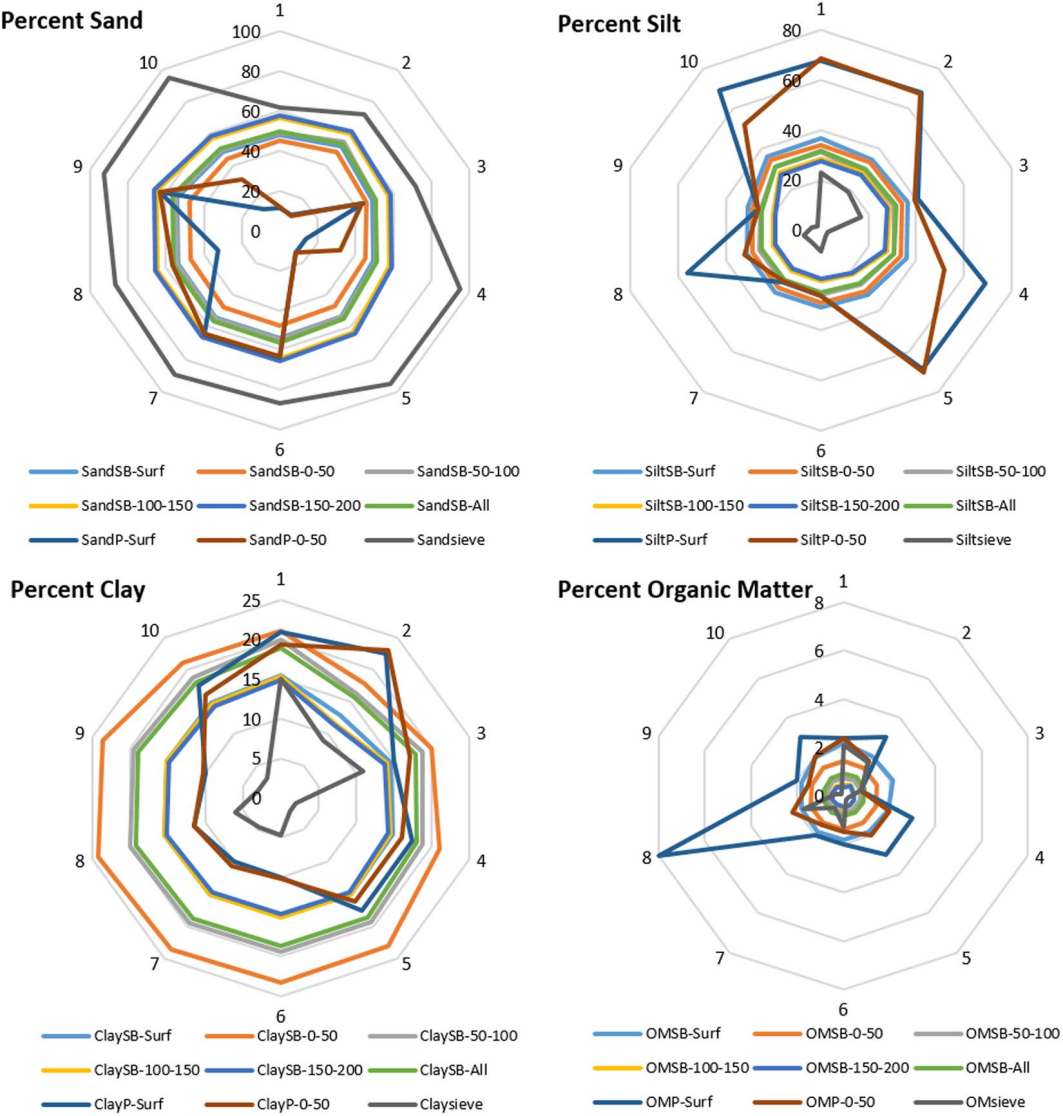
Soil textural compositions of the soils at the ten sites. SB-Surf (subbasin surface level), SB-0–50 (0–50 cm of subbasin), SB-50–100 (50–100 cm of subbasin), SB-100–150 (100–150 cm of subbasin), SB-150–200 (150–200 cm of subbasin), SB-All (0–200 cm of subbasin), P-Surf (point surface), P-0–50 (point 0–50 cm), Sieve (sieve analysis 0–30 cm of streambed).

**Figure 3:**
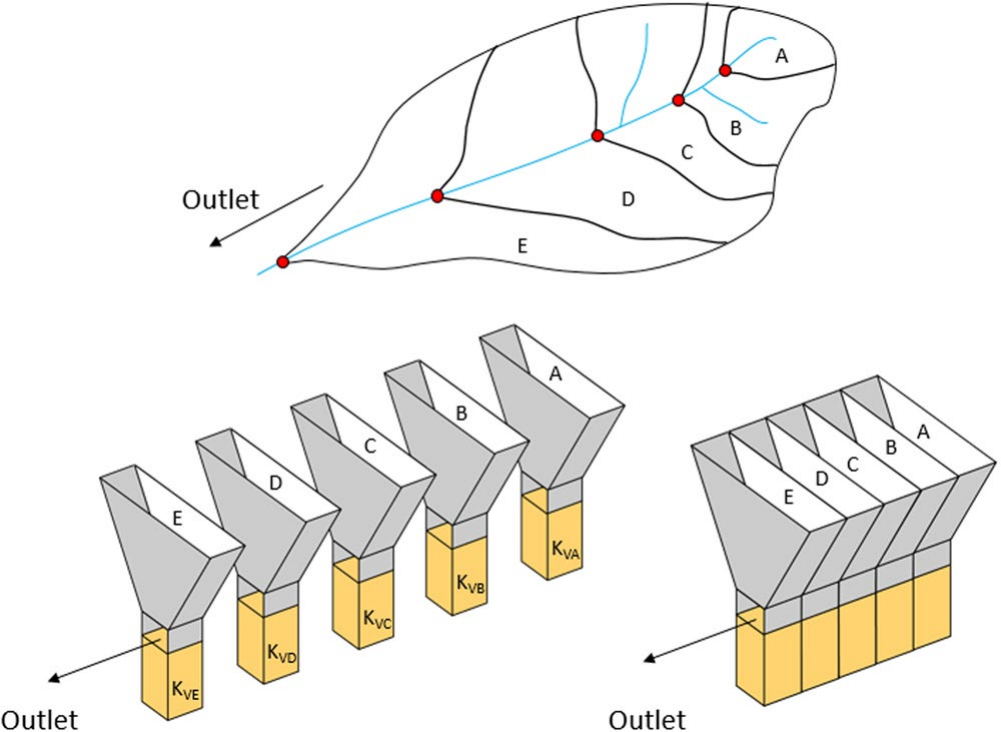
Breakdown of the MSNF approach. *Top*: A typical nested watershed; *Bottom left*: Five separate funnels representing five sub-watersheds; *Bottom right*: Funnels coupled to form a MSNF.

Spatial distributions of soil texture show that, compared to the upland areas, there is about twice more silt than sand in the downstream areas (Sites 4 and 10) of the watershed (Fig. 1). Conversely, *in situ* permeameter tests and sieve analysis show that the streambed is predominantly sandy (>95%) in the downstream areas with higher *K*_*v*_ values (Fig. 2). In general, we observe that geometric mean *K*_*v*_ values tend to increase in the downstream direction in the study area. This seems counterintuitive because *K*_*v*_ is expected to decrease going downstream since the grain size of streambed sediments typically decreases with distance downstream due to abrasion, sorting, and selective transport^40^. However, a downstream transition occurs in stream channels due to the assortment of sediments coming from all points in a watershed and the spatial variation of soil textural properties of the sediment. In addition, the sediment source of the tributaries plays a major role in controlling the grain-size distribution for streambed sediments^41^. A downstream decrease in *K*_*v*_ would be true only if all the sediments enter a stream only from the upper headwaters and there are no sediment contributions from tributaries, streambank erosion and runoff from adjacent fields that enters the stream by flowing over the streambanks. In reality, storms deliver large amounts of eroded sediment from the surrounding landscape. Therefore, we introduce a new approach called Multi-Stemmed Nested Funnel (MSNF) to capture the effect of the spatial variability of soil, reach and watershed properties on *K*_*v*_ prediction (Fig. 3).

### The multi-stemmed nested funnel (MSNF) approach

MSNF approach is based on the concept of nested hierarchy of lower-ordered sub-watersheds and also involves the understanding of soil erosion and sediment mobilization processes. A large watershed is typically made up of many sub-watersheds that are drained by tributary streams and rivers. These sub-watersheds, in turn, are also made up of smaller watersheds of streams draining into their main channels. Headwater watersheds are known to be major contributors of sediments to downstream reaches. This approach suggests that the order of magnitude of *K*_*v*_ (m/day) depends on the textural composition of the sediments coming from headwater watersheds as well as reach and watershed attributes. Thus we hypothesize that a point estimate of *K*_*v*_ should be a function of the sediments coming from the entire area it drains, the reach properties as well as the contributing watershed attributes. Also, point estimates should vary across multiple stream orders due to the heterogeneity and anisotropy of streambed materials.

We choose seven frequently available watershed and soil characteristics (SSURGO datasets) as predictor variables for this study. These are drainage area (*DA*), reach slope (*Rch_Slp*), percent organic matter (*OM*), percent sand, percent silt, percent clay and soil erodibility factor (*K_Erod*). Although other watershed-scale and reach-scale attributes derived from digital elevation models (DEMs) such as the watershed elevations, reach elevations, reach length, width at top of bank, depth, width-depth ratio, and average width of tributary channels would also affect *K*_*v*_, we do not use them in this study. This is partly because of their high sensitivity to spatial resolutions of DEMs used in most studies. These attributes are major inputs to distributed parameter watershed models that are used in simulating the hydrologic response of a watershed^42,43^. Moreover, with 93 permeameter tests carried out across 10 sites (n = 10), such sample size with many predictor variables will lead to overfitting which reduces the accuracy of the estimates and the power of the PTFs.

**Figure 4:**
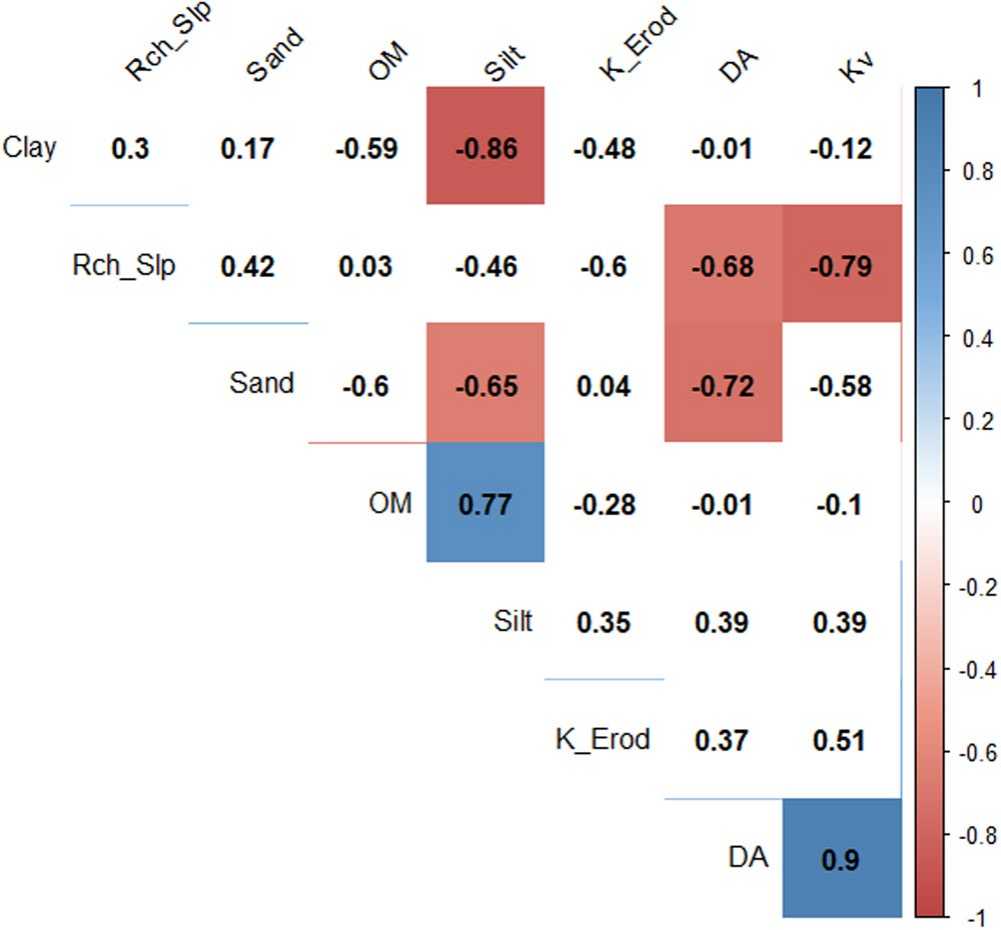
Correlation matrix of all variables. The correlation between *K*_*v*_ and *DA* is highly positive while *K*_*v*_ and *Rch_Slp* are highly negatively correlated.

The correlation matrix shows that *K*_*v*_ is more highly correlated with *DA* than with other selected predictor variables (Fig. 4). There is also a negative correlation between *DA* and *Rch_Slp*. Several studies related to streamflow and sediment yield have also shown a negative relationship between drainage area and average watershed slope^44–46^. Although the correlation coefficients of streambed *K*_*v*_ with *OM* and clay are relatively small, both *OM* and clay are still suitable for developing PTFs because of the relatively high inter-correlation among soil textural properties.

### Developing PTFs for predicting *K*_*v*_

**Figure 5:**
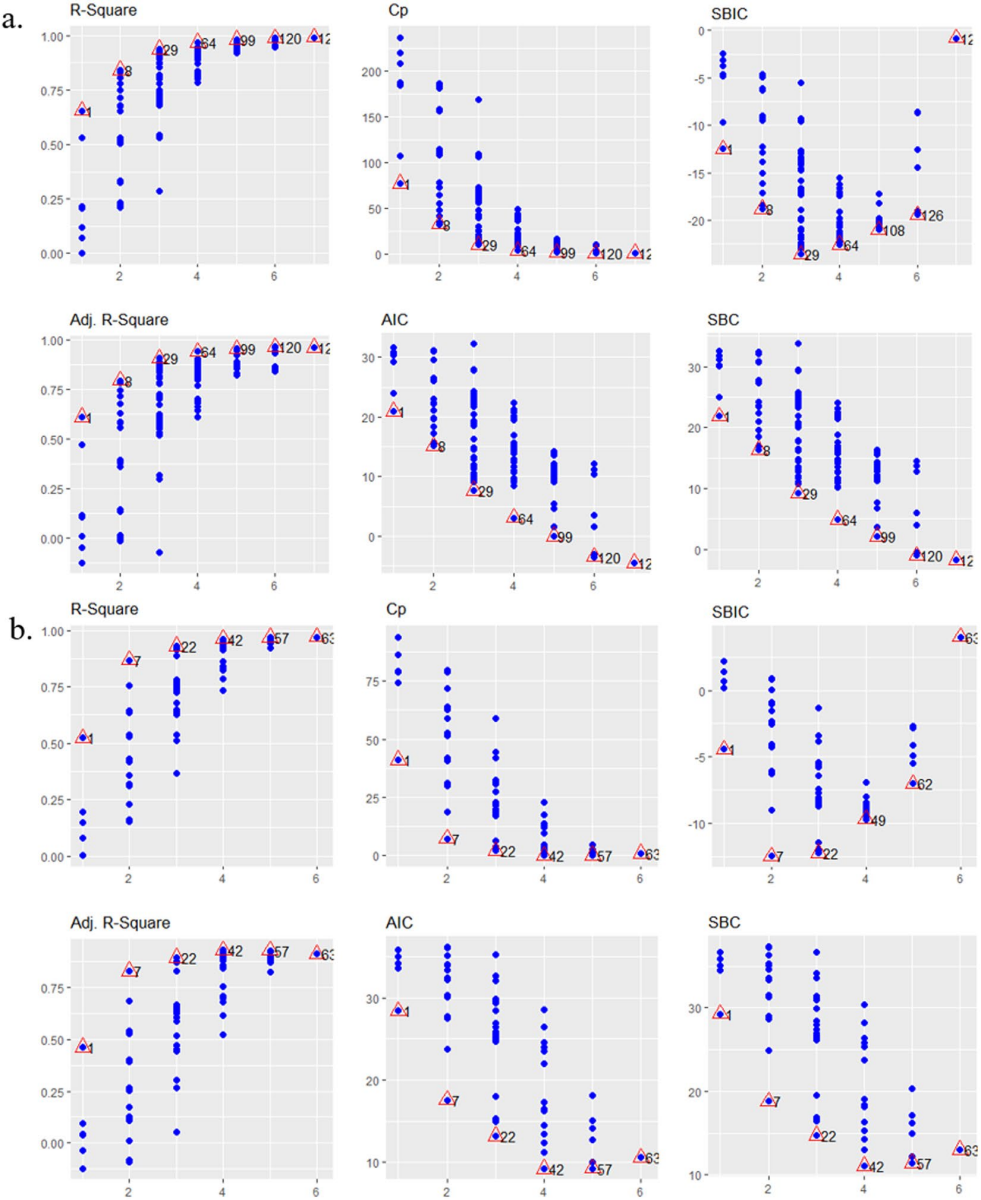
Pedo-transfer functions. (**a)**, All possible PTFs for predicting *Log*(*K*_*v*_). The triangles indicate the best subset PTFs and the overall model numbers. (**b)**, All possible PTFs for predicting *Log*(*K*_*v*_*DA)*. The triangles indicate the best subset PTFs and the overall model numbers.

With the MSNF approach and seven predictor variables, all 127 possible PTFs based on all possible combinations of these variables were developed and analyzed. The performances of all PTFs were assessed by the values of thirteen selection criteria (see Methods). The distributions of all the possible k-variable (k = 1, 2, …, 7) models for six criteria are shown (Fig. 5a). The best subset regression models were selected for each variable-number category using these criteria. That is, of all the possible k-variable models, the best performing model was selected. Since there is only one possible model with seven variables (k = 7), this implies that this is the best 7-variable model.

Except for the best Model 4 (k = 4), all the best subset PTFs consist of *DA* as a predictor and explains 65% of the variance in *LogKv*. The next most important variable is *OM*. Results indicate that there is no consistency in the selection of the overall best PTF based on these thirteen criteria, although Model 5 appears to be the strongest candidate since five out of the thirteen selection criteria choose Model 5 as the overall best PTF.

### *K*_*v*_*DA* as a better response variable

In order to better predict *K*_*v*_ spatially, there is a need to avoid seeking exactness where only an approximation is possible, and accept the degree of imprecision that the nature of complex erosion and sedimentation processes allow. Therefore, we introduce a new response variable (*K*_*v*_*DA*) for predicting the order of magnitude of variation of *K*_*v*_ so as to help balance the accuracy we seek with the uncertainty that exists. While *K*_*v*_ is an intrinsic property of streambed materials, *K*_*v*_*DA* is an extrinsic property which behaves like hydraulic conductance and is a property of a reach which varies depending upon its shape and size as well as watershed attributes. It is important to note that whereas hydraulic conductance is the product of hydraulic conductivity, stream width and length of stream reach divided by the thickness of streambed, *K*_*v*_*DA* is the product of streambed vertical hydraulic conductivity at a location and the watershed area drained by that location.

Coupling *K*_*v*_ and *DA* can be important for reducing the fuzziness in the selection of the overall best PTF. It can also help to capture the hydrological processes which control magnitude of variation of *K*_*v*_ across scale, soil texture domains, as well as across different stream orders. Based on the MSNF approach, *K*_*v*_*DA* is used as a response variable that is dependent on the long-term sediment transport and deposition processes by overland flow from the surrounding landscape. Figure 5a shows all the 63 possible PTFs for predicting log-transformed *KvDA* (that is *LogKvDA*) from percent OM, sand, silt and clay content as well as *Rch_Slp* and *K_Erod*.

**Figure 6:**
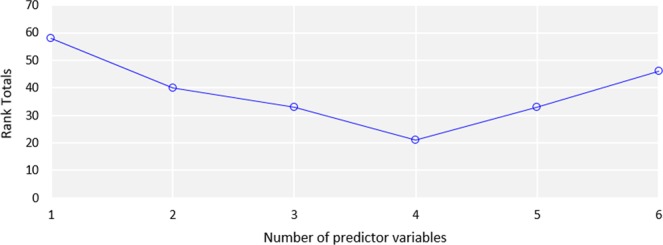
Rank totals of the best subset regression models for predicting *Log*(*K*_*v*_*DA)*.

In contrast to PTFs for predicting *LogKv*, different model categories select *LogRch_Slp* and *LogOM* as the most significant predictor variables when *K*_*v*_ is coupled with *DA*. Models 1, 5, and 6 select *LogRch_Slp* as the best predictor variable while Models 2, 3 and 4 select *LogOM*. Comparison of the performance of the best subset PTFs and the rank totals of the selection criteria (Fig. 6) shows that Model 4 is the overall best PTF due to a better consistency of all the selection criteria. This implies that *OM*, sand, silt and clay contents are the best predictor variables for predicting *LogKvDA*.

## Conclusion

Beyond demonstrating that using *K*_*v*_*DA* as the response variable in the MSNF approach enables prediction of streambed *K*_*v*_ with lower prediction error (when compared to using *K*_*v*_ as the response variable), it is also important to note that predicted *K*_*v*_ values are only rough estimates and are probably best considered as order-of-magnitude estimates. In many applications of distributed models, we believe the MSNF approach we propose can be useful in hydrological modeling using tools such as Soil and Water Assessment Tool (SWAT) and MODFLOW. Prediction errors for the “best” PTF can be used to determine the minimum and maximum *K*_*v*_ values for calibration purposes. However, it is important to note that the use of different prediction error equations in predicting calibration ranges should be expected to lead to different results in terms of the predicted uncertainty limits. We hope that the MSNF approach might provide the basis for further studies which include more soils from the soil texture triangle, specifically clay. Our results, which shall be corroborated by further studies, support the importance of using spatial *K*_*v*_ in modeling to understand the interactions between streams and aquifers.

## Methods

### Study area

Our study site is located in southwest Nebraska and eastern Colorado, USA. It is a sub-watershed of the Republican River watershed. Like much of the High Plains aquifer, Frenchman Creek watershed has experienced significant reductions in groundwater levels and streamflow over the past five decades. The water table has declined ~25 m and the perennial reach of the stream has shortened by more than 21 km. The major tributaries include Stinking Water Creek, Spring Creek, and Sand Creek.

### Data collection

In this study, ninety three *in-situ* falling head permeameter tests were carried out at 10 test sites within the Frenchman Creek watershed (Fig. 1). Seven sites (Sites 4–10) are on Frenchman Creek, two sites (Sites 2 and 3) are on Stinking Water Creek and one site (Site 1) on Spring Creek. The number of sites was limited to landowner access. Other tributaries within the watershed were dry at the time of the study. Each test site comprised of at least three transects and each transect comprised of at least three *K*_*v*_ measurements. Transparent tubes (76 cm long and 8 cm inside diameter or 183 cm long and 6.8 cm inside diameter) were pressed vertically into the channel sediments. The thickness of the tube wall is about 3 mm, typical of many previous studies^18,28^. The locations of permeameter stations were mapped with a global positioning system (GPS). For each *K*_*v*_ measurement, the tube was inserted into the stream bed to a depth of 30 cm. Before beginning the falling head test, it was assumed the stream water level was equal to the groundwater level. This assumption increased measurement uncertainty, but likely by a small amount compared to the variability within and between sites. The surface water-level at the streambed surface was considered as the initial hydraulic head at the measurement point. Water was added slowly to fill up the tube from the top so as not to disturb the sediments inside the tube. As the hydraulic head in the tube began to fall, a series of hydraulic heads at given times were recorded for the derivation of the vertical hydraulic conductivity of the sediment column. *K*_*v*_ (m/day) was calculated using Eq. (1) derived from Hvorslev’s^23^ equation.

1 $${K}_{v}=\frac{\frac{\pi D}{11m}+{L}_{v}}{t}ln(\frac{{H}_{0}}{H})$$

where *H* is the water level inside the permeameter relative to the ambient pre-test water level, *D* is the inside diameter of the tube, *L*_*v*_ is the length of the sediment in the tube, *t* is time, and *m* is the isotropic transformation ratio $\sqrt{{K}_{h}/{K}_{v}}$ where *K*_*h*_ is the horizontal hydraulic conductivity of the sediment around the base of the tube. This study used the average of *K*_*v*_ values using *m* = 1 and *m* = ∞. Note that the m = ∞ scenario simplifies to the standard Darcy equation^24^.

### Data analysis

To check whether the distribution of *K*_*v*_ are normal for the sites, formal tests of normality were carried out using six normality tests^47^. Anderson-Darling (AD), Cramer-von Mises (CVM), Lilliefors (LL), Pearson chi-square (CSQ), Shapiro-Francia (SF), and Shapiro-Wilk (SW) tests were applied at 0.05 significance level. Owing to the fact that there are contradicting results as to which test is the optimal or best test^48^, these six normality tests were compared in order to see how they performed for both non-transformed and log-transformed *K*_*v*_ values.

For more general heterogeneous systems, the effective hydraulic conductivity is known to be the geometric mean since samples of hydraulic conductivity in most cases follow lognormal distribution. Since *K*_*v*_ values may vary by orders of magnitude within a short distance along a river reach and across a section, the geometric mean for each site was used in this study to capture the spatial variation.

The database used in this study includes soil datasets from the SSURGO database that consists of information about soils as collected by the National Cooperative Soil Survey^49^ in the Unites States. Spatial soil properties (% sand, silt, clay and organic matter) at 0–50 cm depth, *K_Erod* (fraction), *Rch_Slp* (fraction) and *DA* (ha) were extracted using Soil Map Viewer in ArcMap 10.3.

PTFs for predicting the order of magnitude of *K*_*v*_ were developed using a new approach called Multi-Stemmed Nested Funnel (Fig. 3). A new response variable (*KvDA*) was also introduced to help in the selection of the most significant predictor variables (Fig. 5b). Multiple linear regression (MLR) method was used to develop the PTFs because it has been used extensively due to its simplicity, good application and accuracy. The PTFs were developed by log-transforming all the variables in order to avoid heteroscedasticity and non-normality of the residuals of the regressions. We evaluated and compared the predictive performance of all possible 127 PTFs using thirteen performance indicators including R-squared (R^2^), Adjusted R-squared (Adj.R^2^), Mallow’s C(p), Akaike Information Criteria (AIC), corrected Akaike Information Criteria (AICc), Sawa’s Bayesian Information Criteria (SBIC), Schwarz Bayesian Criteria (SBC), Mean Squared Error of Prediction (MSEP), Final Prediction Error (FPE), Hocking’s Sp (HSP), Amemiya Prediction Criteria (APC), Leave-One-Out Cross-Validation (LOOCV), and Predicted Residual Error Sum of Squares (PRESS). The major difference between these criteria is how severely each penalizes increases in number of predictor variables (Eqs. (2–14)). For good prediction, the overall “best” PTF is the one that maximizes R^2^, Adj. R^2^ and APC and minimizes the other selection criteria. All statistical analyses and calculations were done using R version 3.4.4^50^.

2 $${R}^{2}=\frac{{\sum }_{i}\,{({\hat{x}}_{i}-\bar{x})}^{2}}{{\sum }_{i}\,{({x}_{i}-\bar{x})}^{2}}$$

3 $$Adj.{R}^{2}=1-\frac{(1-{R}^{2})(N-1)}{N-(p+1)}$$

4 $$C(p)=\frac{SS{E}_{p}}{{S}^{2}}-N+2p$$

where $SS{E}_{p}=\mathop{\sum }\limits_{i=1}^{N}\,{({x}_{i}-{\hat{x}}_{ip})}^{2}$ is the error sum of squares for the model with *p* explanatory variables

5 $$AIC=N\,ln(\frac{SS{E}_{p}}{N})+2p$$

6 $$AI{C}_{C}=AIC+\frac{2p(p+1)}{N-(p+1)}$$

7 $$SBIC=N\,ln(\frac{SS{E}_{p}}{N})+\frac{2(p+2)N{\sigma }^{2}}{SS{E}_{p}}-\frac{2{N}^{2}{\sigma }^{4}}{SS{{E}_{p}}^{2}}$$

8 $$SBC=N\,ln(\frac{SS{E}_{p}}{N})+p\,ln(N)$$

9 $$MSEP=(\frac{SS{E}_{p}}{N-{p}^{\ast }})(n+1)(n-2)/n(n-{p}^{\ast }-1)$$

10 $$FPE=(\frac{SS{E}_{p}}{N-{p}^{\ast }})(\frac{N+{p}^{\ast }}{N})$$

11 $$HSP=\frac{SS{E}_{p}}{(N-{p}^{\ast })(N-{p}^{\ast }-1)}$$

12 $$APC=(\frac{N+{p}^{\ast }}{N-{p}^{\ast }})(1-{R}^{2})$$

13 $$LOOCV=\frac{1}{N}\mathop{\sum }\limits_{i=1}^{N}\,{({x}_{i}-{\hat{x}}_{ii})}^{2}$$

14 $$PRESS=\mathop{\sum }\limits_{i=1}^{N}\,{({x}_{i}-{\hat{x}}_{ii})}^{2}$$

where ${x}_{i}$ is the observed value, ${\hat{x}}_{i}$ is the predicted value, $\bar{x}$ is the observed mean value, $p$ is the number of explanatory variables, ${\hat{x}}_{ip}$ is the predicted value of the *i*th observation of *x* from the *p* explanatory variables, ${p}^{\ast }$ is the number of explanatory variables including the intercept, ${\hat{x}}_{ii}$ is the predicted value of the *i*th observation of *x* using all data except *i*th, ${S}^{2}$ is the residual mean square after regression on the complete set of *K* explanatory variables, *N* is the sample size, *L* is the maximized value of the likelihood function for a model, ${\sigma }^{2}$ is the pure error variance fitting the full model.

In order to select the overall “best” PTF for predicting *LogKvDA*, all the best subset PTFs (i.e. best *n*-predictor models) are selected for each number of predictor variables. For each criterion, the models are ranked from 1 to *n*, and the rank total for each best *n*-predictor model is calculated by adding all the ranks for all the criteria. All rank totals exclude R^2^ because it increases every time a predictor variable is added to a model, even if due to chance alone. If a model has too many predictor variables, it begins to model the random noise in the data. Hence, this overfitting produces misleadingly high R^2^ values and a reduced ability to make predictions. Also, to avoid bias due to double counting, LOOCV was excluded in calculating the rank totals because it is identical to the PRESS.

## Data Availability

The datasets generated during and/or analysed during the current study are available from the corresponding author on reasonable request.
